# Survival outcome according to KRAS mutation status in newly diagnosed patients with stage IV non-small cell lung cancer treated with platinum doublet chemotherapy

**DOI:** 10.18632/oncotarget.4711

**Published:** 2015-09-03

**Authors:** Anna K. Brady, Jonathan D. McNeill, Brendan Judy, Joshua Bauml, Tracey L. Evans, Roger B. Cohen, Corey Langer, Anil Vachani, Charu Aggarwal

**Affiliations:** ^1^ Department of Medicine, Washington University Medical Center, St. Louis, MO, USA; ^2^ Ruth and Raymond Perelman School of Medicine of the University of Pennsylvania, Philadelphia, PA, USA; ^3^ Jefferson Medical College, Thomas Jefferson University, Philadelphia, PA, USA; ^4^ Division of Hematology and Oncology, University of Pennsylvania, Philadelphia, PA, USA; ^5^ Division of Pulmonary, Allergy and Critical Care, University of Pennsylvania, Philadelphia, PA, USA

**Keywords:** KRAS, non-small cell lung cancer, bevacizumab

## Abstract

**Introduction:**

Mutations (MT) of the *KRAS* gene are the most common mutation in non-small cell lung cancer (NSCLC), seen in about 20–25% of all adenocarcinomas. Effect of KRAS MT on response to cytotoxic chemotherapy is unclear.

**Methods:**

We undertook a single-institution retrospective analysis of 93 consecutive patients with stage IV NSCLC adenocarcinoma with known KRAS and EGFR MT status to determine the association of KRAS MT with survival. All patients were treated between January 1, 2008 and December 31, 2011 with standard platinum based chemotherapy at the University of Pennsylvania. Overall and progression free survival were analyzed using Kaplan-Meier and Cox proportional hazard methods.

**Results:**

All patients in this series received platinum doublet chemotherapy, and 42 (45%) received bevacizumab. Overall survival and progression free survival for patients with KRAS MT was no worse than for patients with wild type KRAS. Median overall survival for patients with KRAS MT was 19 months (mo) vs. 15.6 mo for KRAS WT, *p* = 0.34, and progression-free survival was 6.2 mo in patients with KRAS MT vs. 7mo in patients with KRAS WT, *p* = 0.51. In multivariable analysis including age, race, gender, and ECOG PS, KRAS MT was not associated with overall survival (HR 1.12, 95% CI 0.58–2.16, *p* = 0.74) or progression free survival (HR 0.80, 95% CI 0.48–1.34, *p* = 41). Of note, receipt of bevacizumab was associated with improved overall survival only in KRAS WT patients (HR 0.34, *p* = 0.01).

**Conclusions:**

KRAS MT are not associated with inferior progression-free and overall survival in advanced NSCLC patients treated with standard first-line platinum-based chemotherapy.

## INTRODUCTION

Our understanding of the biology of NSCLC has accelerated dramatically with the recognition that oncogenic driver mutations often play a decisive role in prognosis and treatment response. The introduction of molecular markers is transforming the treatment paradigms for this disease [1–4]. One of the most common molecular changes in NSCLC is mutations in KRAS, usually point mutations in codon 12 or 13. KRAS is a member of the RAS family of oncogenes that encode small GTPases involved in cellular signal transduction. KRAS mutations (MT) are more common in adenocarcinoma than in other NSCLC histologies [5]. KRAS MT occur more often in smokers than nonsmokers [6–9] unlike EGFR mutations and ALK translocations. Targeted therapies, including tyrosine kinase inhibitors (TKIs), for EGFR and EML-4ALK mutations have improved progression-free survival in patients bearing the relevant mutations [1–3]. Despite initial forays [10] into targeted therapy for KRAS MT NSCLC, there are no such approved agents at this time.

Much of the existing literature suggests that NSCLC patients with KRAS MT have inferior outcomes compared to those with KRAS WT [7, 11–13]. This literature has a number of serious limitations, however, including small sample sizes and patient heterogeneity. A number of studies have analyzed patient series comprising a mix of patients with different NSCLC histologies (adenocarcinoma and squamous cell carcinoma) and stages [7, 11, 13–18].

The predictive value of KRAS mutations in NSCLC for therapy selection also remains unclear [19, 20]. Various reports have suggested that treatment with TKIs such as erlotinib results in inferior outcomes in patients with KRAS MT NSCLC, [12, 21] but this view is not universal [22]. While KRAS MT status is clearly associated with lack of response to the anti-EGFR antibody cetuximab in colorectal cancer, [23] in lung adenocarcinoma KRAS MT status does not appear to predict response to this agent [24].

Similarly, there are reports suggesting an adverse effect of chemotherapy in patients with KRAS MT compared to KRAS WT [20]. For the most part the series are small and cover diverse therapeutic settings (adjuvant therapy [14] and therapy of stage IV disease) and some of the older studies used older chemotherapy regimens such as cisplatin and vinorelbine.

In the absence of targeted therapies, and personalized approaches, platinum doublet chemotherapy (with or without bevacizumab) remains the current standard of care for these patients. Given persistent uncertainty about the predictive value of KRAS MT status in NSCLC, we performed a retrospective, single-institution study to determine the relationship between KRAS MT and survival after platinum-based chemotherapy in patients with stage IV lung adenocarcinoma.

## RESULTS

### Study population and frequency of KRAS mutations

Median age was 60 years; 51% were women and 20% were lifelong nonsmokers. Baseline characteristics were similar in both groups (Table 1). Thirty-eight (40%) patients had KRAS MT (See Table 2). Of the 55 KRAS wild type (WT) subjects, 5 (5%) had EGFR MT, and 50 (53%) were EGFR WT. Most patients had an ECOG performance status of 0 or 1 (34% and 49%, respectively). The majority of KRAS MT were codon 12 mutations with 2 codon 13 mutations and 1 not specified. Gly12Cys was the most common amino acid substitution (15 patients), followed by Gly12Val (11 patients). KRAS MTs were more common in current or previous smokers compared to lifelong nonsmokers (47% vs 16% *p* = 0.01).

**Table 1:** Baseline characteristics of study population

	KRAS WT (*N* = 55)	KRAS MT (*N* = 38)	*p*-value
**Age, mean (SD)**	58.0 (±11.6)	62.8 (±11.8)	*P* = 0.06
**Sex, n(%)** Male Female	31 (56) 24 (43)	15 (39) 23 (61)	*p* = 0.11
**Race, n(%)** White African-American Asian	45 (82) 8 (15) 2 (4)	34 (89) 4 (11) 0 (0)	*p* = 0.60
**Smoking status, n(%)** Never Former Current	16 (29) 27 (49) 12 (22)	3 (8) 23 (60) 12 (32)	*p* = 0.04
**Pack-years, mean (SD)**	37.0 (23.5)	34.0 (26.9)	*p* = 0.62
**ECOG performance status, n(%)** 0 1 2	18 (32) 30 (55) 7 (13)	15 (39) 15 (39) 8 (21)	*p* = 0.34
**Platinum chemotherapy, n(%)** Carboplatin Cisplatin	53 (96) 2 (4)	36 (95) 5 (5)	
**Pemetrexed, n(%)**	42 (76)	31 (82)	*p* = 0.55
**Bevacizumab, n(%)**	26 (47)	16 (42)	*p* = 0.62

**Table 2:** KRAS and EGFR mutation status

	*N*	Percent
KRAS MT	38	41%
Codon 12	35	
Codon 13	2	
EGFR MT	5	5%
KRAS WT and EGFR WT	49	53%

### Treatment

All subjects received first-line platinum doublet chemotherapy; 96% received carboplatin and the remainder cisplatin. A majority of patients (78%) received pemetrexed as the platinum partner; 15 patients (16%) received either paclitaxel or docetaxel (Table 1). Nearly half (45%) the subjects received bevacizumab in addition to the platinum doublet. Patients with KRAS MT were as likely as patients without KRAS MT to receive pemetrexed or bevacizumab based chemotherapies (Table 1).

### Effect of KRAS on overall survival and progression-free survival

Among 47 patients who were still alive at the close of the study, median length of follow-up was 30 months (range 2–51 months). There were 46 (49%) deaths during the study period, with a median OS for all patients of 19.0 months (95%CI, 14.–28.9 months). There was no significant difference in OS between patients with KRAS WT and KRAS MT (median OS 19 months vs. 15.6 months, *p* = 0.34; Figure 1A). There were a total of 81 (90%) patients with progression during the study period, with a median PFS for all subjects of 6.9 months (95%CI, 4.9 to 9.3 months). There was no significant difference in PFS between patients with KRAS WT and KRAS MT (median PFS 6.2 mo vs. 7.0 mo; *p* = 0.51; Figure 1B).

**Figure 1A:**
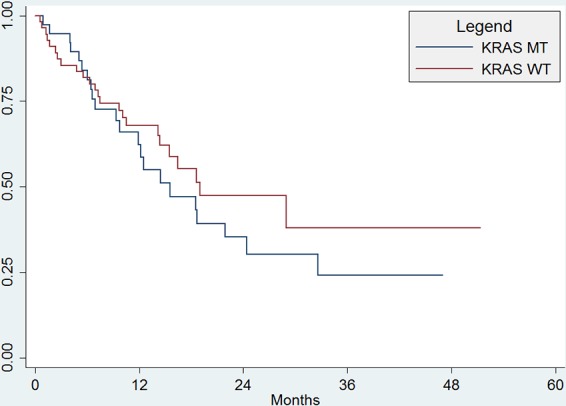
Overall Survival There was no significant difference in OS between patients with KRAS WT and KRAS MT (median OS 19 months vs. 15.6 months, *p* = 0.34).

**Figure 1B:**
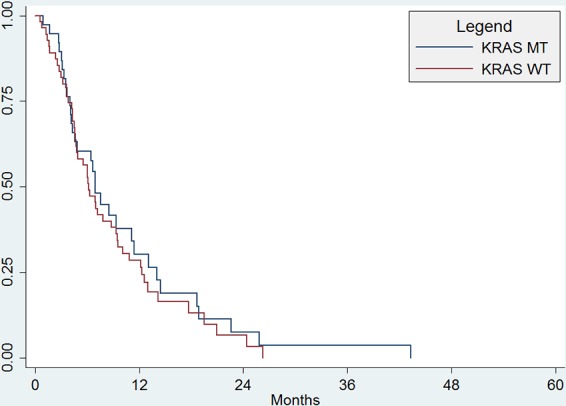
Progression-free survival There was no significant difference in PFS between patients with KRAS WT and KRAS MT (median PFS 6.2 mo vs. 7.0 mo; *p* = 0.51).

Sex, age, and tobacco history were not associated with overall survival or PFS in univariate analyses (Table 3 for OS, data for PFS not shown). There was, however, significantly longer OS among patients with an ECOG performance status of 0 or 1 compared to patients with a performance status of 2 (median OS 28.9 months vs. 14.2 months; *p* = 0.01). In multivariable analysis controlling for age, race, gender, and ECOG PS, there was no significant difference in OS (HR 1.12, 95% CI 0.58–2.16, *p* = 0.74) or PFS (HR 0.80, 95% CI 0.48–1.34, *p* = 41) between patients with KRAS MT and those with KRAS WT.

**Table 3:** Overall Survival

		Unadjusted model		Multivariable model	
Demographic or Characteristic	No. of Patients (*n* = 93)	Median OS (95% CI)	*P* value	HR (95% CI)	*P* value
**Gender**					
Male	46	16.4 (10.1–32.6)	0.25	Ref.	-
Female	47	19.0 (12.5–xx)		0.81 (0.42–1.55)	0.52
Age	93	NA	NA	1.02 (0.99–1.06)	0.12
**Race**					
White	79	18.5 (12.5–28.9)	0.83	Ref.	-
Black/Other	14	Not Reached		0.58 (0.21–1.61)	0.20
**ECOG PS**					
0	33	28.9 (18.5–xx)	0.0003	Ref.	-
1	45	19.0 (9.8–xx)		1.93 (0.94–3.93)	0.07
2	15	6.4 (1.7–14.5)		4.64 (1.97–10.9)	<0.0001
**KRAS**					
WT	55	19.0 (14.2–xx)	0.34	Ref.	
MT	38	15.6 (9.8–24.4)		1.12(0.58–2.16)	0.74

### KRAS status and differential efficacy of bevacizumab

Nearly half (45%) of the subjects received bevacizumab. We evaluated the effect of bevacizumab therapy to determine whether outcomes varied by KRAS mutation status. Among KRAS WT patients, the use of bevacizumab in addition to platinum doublet therapy resulted in significant improvements in OS (median 28.9 mo vs. 14.2 mo; *p* = 0.01) and PFS (median 9.5 mo vs. 4.8 mo, *p* = 0.004). This effect remained significant for KRAS WT patients in multivariable analysis controlling for age, race, gender, and ECOG PS (HR 0.34, 95% CI 0.13–0.94 for OS and HR 0.37, 95% CI 0.19–0.72 for PFS), but was not seen in patients with KRAS MT (HR 0.88, 95%CI 0.30–2.76 for OS and HR 0.96, 95% 0.35–2.62 for PFS).

## DISCUSSION

We studied a consecutive series of patients with stage IV NSCLC adenocarcinoma and known EGFR and KRAS mutation status. In our population, KRAS MTs were common, similar to what has been observed in other series. As expected, KRAS MTs were more frequent in smokers and were nearly all codon 12 mutations. Our key finding is that KRAS MTs were not associated with OS or PFS when controlling for age, smoking status, and ECOG PS in a relatively uniform population of patients with stage IV adenocarcinoma receiving platinum doublet chemotherapy. In addition, we found that bevacizumab use was associated with significantly better outcome in the KRAS WT population, but not in those with KRAS MTs. A similar effect was observed in a study of neoadjuvant bevacizumab, where 10 patients with KRAS MT NSCLC who underwent resection did not have a major pathological response [27].

KRAS is the most frequently mutated oncogene in patients with lung adenocarcinoma, and many studies have been conducted to evaluate its clinical and therapeutic implications. Despite the successes with targeted therapy for driver mutations, personalized therapy for patients with KRAS MT is still under development. Platinum doublet chemotherapy, with or without bevacizumab, is the standard of care.

The predictive role of KRAS is controversial, with prior studies of KRAS MT in lung adenocarcinoma yielding contradictory conclusions. In the adjuvant setting, the relationship of KRAS MT to chemotherapy was explored in a molecular analysis of the patients included in the JBR.10 clinical trial. In this analysis, KRAS MT were neither prognostic of survival nor predictive of a differential benefit from adjuvant cisplatin and vinorelbine [15]. Shepherd et al reached similar conclusions in their exploratory analyses characterizing relationships between KRAS MT and survival outcomes across three adjuvant trials from the LACE BIO meta-analysis. They analyzed 300 patients with KRAS MT (predominantly codon 12 mutations). For the patients that received adjuvant chemotherapy, no significant benefit was observed for KRAS WT or patients with codon-12 KRAS MT. However, for patients with codon 13 KRAS MT, adjuvant chemotherapy was associated with a worse survival. Since the number of patients with codon 13 MT was small, the authors concluded that KRAS status could not be recommended to select patients with NSCLC for adjuvant chemotherapy [14].

Furthermore, retrospective studies in the metastatic setting have shown mixed chemotherapy effect based on the presence or absence of a KRAS MT [28, 29]. Sun et al [20] reported a worse response rate and PFS for the pemetrexed-based regimen in KRAS MT compared to KRAS WT subjects; KRAS MT were also associated with inferior outcomes after gemcitabine-based chemotherapy, but there was no difference in KRAS MT and WT patients receiving a taxane-based regimen.

In contrast to the previous studies, we did not find a worse survival outcome for patients with KRAS MT. Majority of our patients had codon 12 MT, and were treated with a pemetrexed based regimen, making comparisons between different chemotherapy regimens difficult. Our study adds to the above literature by exploring the relationship of KRAS MT to survival in patients with metastatic adenocarcinoma. The strength of our investigation is our relatively homogenous pt population: all patients had stage IV adenocarcinoma, the percentage of KRAS MT is similar to that reported in the literature, all patients received platinum based doublet chemotherapy in 1st line and a majority of patients received a contemporary regimen of carboplatin and pemetrexed. In addition, a significant proportion of the patients received bevacizumab, making this the largest study to explore the relationship of bevacizumab and KRAS MT.

Our study also has several important limitations: first, it is a retrospective study, and inclusion was limited to patients that had been tested for EGFR and KRAS MT. Second, although our study population is large compared to most of the existing literature it is nevertheless relatively small. Third, progression events were determined based on the assessment of the treating oncologist abstracted from the medical record and not by the formal RECIST metrics that might be used in a clinical trial. Fourth, our analysis mainly describes the prognostic role of KRAS codon 12 mutations and provides no insight into the role of KRAS MT codon 13 mutations. Finally, since our patients received a nearly homogenous treatment regimen, we are unable to analyze the effects of different chemotherapy regimens on outcome in patients with KRAS MT compared to KRAS WT NSCLC. However, the high percentage of patients receiving pemetrexed in our study is a notable difference from prior studies [12, 20] which for the most part noted inferior survival for KRAS MT patients receiving systemic chemotherapy with pemetrexed [20]. Based on our observations, it is possible that pemetrexed may have a more favorable effect on KRAS MT tumors than, but our study was not designed to answer this question in a formal manner.

Our study suggests that KRAS MT is not associated with worse PFS and OS in advanced NSCLC patients treated with platinum doublet chemotherapy. Our study suggests that patients with KRAS WT may benefit more from bevacizumab compared to patients with KRAS MT. Due to the small sample size of our patient population, this observation is purely hypothesis generating, and needs further analysis. Our results are not generalizable to earlier-stage or non-adenocarcinoma NSCLC. Based on our observations, patients with Stage IV KRAS MT NSCLC should be treated with similar chemotherapy regimens as KRAS WT patients, including the use of bevacizumab when clinically appropriate. Furthermore, the differential effects of codon 12 and codon 13 KRAS MT should also be analyzed in a larger population; utilizing next generation sequencing in the future would be helpful to explore relationship of different genetic subgroups to overall outcome. In the future, the real utility of KRAS testing will depend on the availability of KRAS directed therapeutics, which has been elusive to date.

## MATERIALS AND METHODS

### Patient population

Consecutive patients diagnosed with Stage IV NSCLC seen at the University of Pennsylvania's Abramson Cancer Center between January 1, 2008 and December 31, 2011 were analyzed retrospectively. Patients with histology other than adenocarcinoma or adenosquamous cancer and patients with stages other than AJCC 7th edition [25] stage IV at presentation were excluded. Patients who did not receive treatment (i.e., were seen only once in consultation) were also excluded, as were those who received no chemotherapy (for example, radiation only) or who received first-line erlotinib. Only patients with known KRAS and EGFR MT status were included. Absence of known EML4-ALK status was permitted given the era in which the study was conducted. Ninety-three patients were included in the final analysis. This study was approved by the Institutional Review Board of the University of Pennsylvania.

### Mutational analysis

Patients were included only if a written report of the mutational analysis could be verified. EGFR and KRAS immunohistochemistry or amplification was not considered. Only EGFR mutations known to be correlated with prognosis (i.e., exon 19 deletion or exon 21 L858R) were considered in our analysis. All KRAS and EGFR mutation testing was done with quantitative polymerase chain reaction. Genomic DNA was extracted from tumor specimens. KRAS mutations in codons 12 and 13 were assessed by direct sequencing of exon 2 using primers for the seven most common point mutations (nucleotides c.34G, c.35G and c.38G) (NM_004985.3). EGFR mutations were assessed by polymerase chain reaction (PCR)-based methods that detect exon 19 deletions and exon 21 leucine-to-arginine codon 858 (L858R) amino acid substitutions [26].

### Clinical data

The following information was collected from the electronic medical record: age at diagnosis, sex, race, ethnicity, smoking status, ECOG performance status at diagnosis; stage at diagnosis (TNM, according to AJCC 7th edition guidelines), histology, method of diagnosis, date of diagnosis; treatment (first, second and third line therapies) and its outcome (response, progression, stable disease), EGFR and KRAS mutation status and whether EML4-ALK mutation testing was performed.

### Statistical analysis

Progression-free-survival (PFS) was calculated from date of diagnosis to date of death or progression; the date of progression was based on documentation by the treating oncologist in the electronic medical record. Overall survival (OS) was calculated from date of diagnosis to date of death or date of last follow-up. Patients were censored at 5/31/2012 or last follow up visit if they were subsequently lost to follow-up prior to the end of data collection. Chi-square analysis was used to describe the relationship of KRAS MT to smoking status (never vs. current or former smoker). Kaplan-Meier analysis was used to estimate overall and progression-free survival. Cox proportional hazard models were used to determine the relationship of KRAS MT to survival, with adjustment for ECOG PS, gender, race, and age. To assess the proportional hazards assumption, we used the Schoenfeld residuals test and complementary log-log plots.
